# Features predicting the success of computerized decision support for prescribing: a systematic review of randomized controlled trials

**DOI:** 10.1186/1472-6947-9-11

**Published:** 2009-02-11

**Authors:** Brent Mollon, Jaron JR Chong, Anne M Holbrook, Melani Sung, Lehana Thabane, Gary Foster

**Affiliations:** 1 The Centre for Evaluation of Medicines, McMaster University, Hamilton, Ontario, Canada; 2 Department of Clinical Pharmacology & Therapeutics, McMaster University, Hamilton, Ontario, Canada; 3 Department of Clinical Epidemiology & Biostatistics, McMaster University, Hamilton, Ontario, Canada

## Abstract

**Background:**

Computerized decision support systems (CDSS) are believed to have the potential to improve the quality of health care delivery, although results from high quality studies have been mixed. We conducted a systematic review to evaluate whether certain features of prescribing decision support systems (RxCDSS) predict successful implementation, change in provider behaviour, and change in patient outcomes.

**Methods:**

A literature search of Medline, EMBASE, CINAHL and INSPEC databases (earliest entry to June 2008) was conducted to identify randomized controlled trials involving RxCDSS. Each citation was independently assessed by two reviewers for outcomes and 28 predefined system features. Statistical analysis of associations between system features and success of outcomes was planned.

**Results:**

Of 4534 citations returned by the search, 41 met the inclusion criteria. Of these, 37 reported successful system implementations, 25 reported success at changing health care provider behaviour, and 5 noted improvements in patient outcomes. A mean of 17 features per study were mentioned. The statistical analysis could not be completed due primarily to the small number of studies and lack of diversity of outcomes. Descriptive analysis did not confirm any feature to be more prevalent in successful trials relative to unsuccessful ones for implementation, provider behaviour or patient outcomes.

**Conclusion:**

While RxCDSSs have the potential to change health care provider behaviour, very few high quality studies show improvement in patient outcomes. Furthermore, the features of the RxCDSS associated with success (or failure) are poorly described, thus making it difficult for system design and implementation to improve.

## Background

Prescribing skills are core to the practice of medicine. As in most developed countries, prescription drugs are currently the fastest growing cost category in Canadian healthcare, exceeding $22 billion annually and increasing at 10.5% yearly [1]. With this increase in medication prescribing follows the potential for adverse drug events, including prescribing errors. It is estimated that medication errors occur in 57 per 1000 orders, with 18.7 – 57.7% of these errors having the potential for harm [2]. The suggestion that detection of preventable errors by health care professionals could improve patient safety and reduce the cost of adverse drug events [3], has been sufficient to spawn a multitude of medication safety initiatives with limited rigorous evaluation of their benefits and harms. Although they have several uses, the main interest in electronic health records (EHR) and computerized decision support systems (CDSS) is to improve patient outcomes by influencing the decision making process of providers [4-6]. CDSS provide patient-specific advice by using algorithms to compare patient characteristics against a knowledge base [7-9]. Prescribing CDSS (RxCDSS) specifically deal with medications and can support basic (e.g. checking for drug-drug interactions) to complex (e.g. integrating patient-specific diagnoses, risk factors, and prior treatments to make a drug recommendation) functions [10]. These systems may include, but do not require, a formal e-prescribing link with pharmacies.

Reviews evaluating the literature surrounding decision support have noted that technologies have the potential to improve practitioner performance, but effects on patient outcomes are still unclear [11-15]. Several features have been linked with successful clinical decision support. These include use of a computer to generate the decision support based on automated EHR data analysis, including provision of recommendations instead of just assessments, and provision of the decision support at the time and location of decision-making and in synchrony with usual clinician workflow [11,12,14,15].

However, only one of these reviews [14] limited their analysis to high quality evidence (randomized controlled trials). None of the reviews systematically separated outcomes by their natural hierarchy of difficulty – system implementation, provider behaviour change and patient outcomes, and focused on features predicting success versus failure for each outcome domain. Finally, while one review was limited to drug order entry systems [14], no study to date has examined all RxCDSS irrespective of the presence of this system feature.

Our objective was to conduct a systematic review of randomized trials, to evaluate the effectiveness of RxCDSS using a hierarchical approach to defining success, and to determine which features of system design or implementation were associated with the success or failure of RxCDSS implementation, change in provider behaviour, and change in patient outcomes.

## Methods

The two primary research questions of this review were: (1) When evaluated rigorously in randomized controlled trials, have current RxCDSS successfully been implemented and altered physician prescribing or patient outcomes? Furthermore, (2) what features of these RxCDSS are associated with success versus failure? Based on the literature and our own experience, we hypothesized that: a) high quality studies of RxCDSS may report successful implementation, but fewer have changed prescriber behaviour and fewer still have demonstrated improved patient outcomes and b) a number of RxCDSS features will be associated with successful versus unsuccessful outcomes as defined above.

### RxCDSS Features

**Figure 1:**
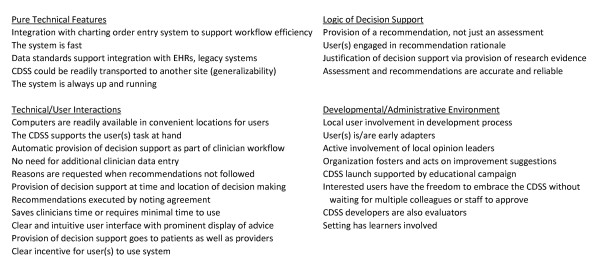
**List of RxCDSS features evaluated**. This figure summarizes the RxCDSS features evaluated in this review.

Potentially important features were identified primarily from our own e-health research program and clinical experience [16] as well as reviews of the literature [11,12,17-19]. A list of 40 features was generated, 12 features were ultimately removed during the review process due to lack of reporting or inability to assess. The remaining 28 features were grouped into 4 categories: Pure technical features, Technical/user interactions, Logic of decision support, and Developmental and Administrative environment (see Figure 1; Additional File 2).

### Study Inclusion/Exclusion

We included reports of RCTs of RxCDSS published in English. We considered a RxCDSS to be an intervention which utilized a computer to analyze patient-specific information to advise a prescriber (primarily a physician) or pharmacist when they were writing or filling a prescription, respectively. Although the decision support itself had to be generated electronically, the support could be delivered by any means (e.g. computer terminal, fax, mail, patient record insert). We only considered systems which intervened before a drug therapy had been chosen by a physician, or had the ability to suggest alternate therapies (i.e. a drug different then that initially prescribed) to be a RxCDSS. These are the more challenging decisions for which to intervene and change. Systems whose sole purpose was to offer 'fine-tuning' advice on a pre-defined therapy – usually dose modification – were not included in this review. Systems primarily focused on diagnosis, vaccination, or nutrition, were also excluded.

### Search Strategy

We searched the databases Medline, EMBASE, CINAHL, and INSPEC for articles published since the earliest entry to June 2008. The detailed search strategy is shown in Additional File 3. The search was individually tailored for each database, with search terms from domains of study methodology, general CDSS terms and RxCDSS identifiers. These included: randomized controlled trial, artificial intelligence, decision support systems, computer-assisted therapy, computerized medical records system, reminder systems, hospital information systems, computer systems, decision support techniques, ambulatory care information systems, computer assisted decision making, medical errors, therapeutic uses, drug therapy, drug information services, drug interactions, drug monitoring, guideline adherence, medication systems, drug administration schedule, drug costs, drug dose-response relationship, and computer assisted drug therapy. A pilot test was completed to ensure that known relevant studies were identified. All citations obtained were downloaded into Reference Manager, version 11.0.

### Study Selection

The titles of all returned studies were reviewed, and those potentially matching our definition of a RxCDSS were kept. Next, the abstracts were assessed independently by two reviewers to determine whether the studies met the inclusion criteria. Disagreements between reviewers were resolved by consensus and, if necessary, by arbitration of a third reviewer. If uncertain whether a study met the inclusion criteria, it moved to the next stage of assessment in order to decrease the likelihood that a relevant study was overlooked.

During full-text review, articles were once again reviewed independently using detailed data extraction forms which extracted details on methods, study validity, study outcomes and features. Before use, the data extraction forms were critiqued for face validity by a panel of methodologists experienced in systematic reviews and CDSS. The forms were also piloted to improve usability.

### Analysis

Methodological quality of each RCT was assessed using a modified scale adapted from Garg *et al *[11]. Our rating system assessed studies on four potential sources of bias: unit of allocation, presence of baseline differences between groups potentially linked to study outcomes, objectiveness of outcome, and completeness of follow-up. Each source of bias was rated on a scale of 0 to 2, with 2 indicating the highest methodological quality. The results of this evaluation were summed with a maximum total possible score of 8.

Study outcomes were assessed for success in each of our three domains of focus: 'Implementation', 'Change in Health Care Provider Behaviour', and 'Change in Patient Outcomes'. Implementation was considered successful if the RxCDSS was successfully introduced and utilized by the clinical staff. A successful change in provider behaviours required reporting of changes such as a decrease in inappropriate prescribing or a change to a more cost-effective therapy. Lastly, impact on patient outcomes was considered successful if the study reported improvements in patients' health (e.g. decreases in morbidity or mortality). These domains of outcomes were hypothesized to be conditional and hierarchical, with success required in implementation before changes in provider behaviour would be noted, and so on. Since the concept of minimal clinically important difference in this area of research remains undefined, outcomes were assessed for statistical significance as reported by the original study [20].

Each RCT report was reviewed several times independently to ensure complete abstraction of features of interest. Each feature on our list was rated for each study as present, absent, or could not assess. 'Could not assess' was used when, even after extensive discussion, reviewers could not agree that a feature was present or absent. For the purposes of analysis, features that could not be assessed were considered absent.

Consensus was obtained as described above for methodological quality scores, RxCDSS success and presence/absence of features. Descriptive statistics were used to characterize the studies included, their degree of success, and the number of features reported. Inter-rater reliability for selected methodological quality score, success and features present or not, was calculated and reported as a kappa statistic. We planned to measure the association between our three-tier definition of success of the individual studies and the feature list using univariate binary logistic regression. This method requires roughly equivalent numbers of successful and unsuccessful studies per outcome. Statistical analyses were conducted using SAS 9.1 (Cary, North Carolina).

## Results

**Figure 2:**
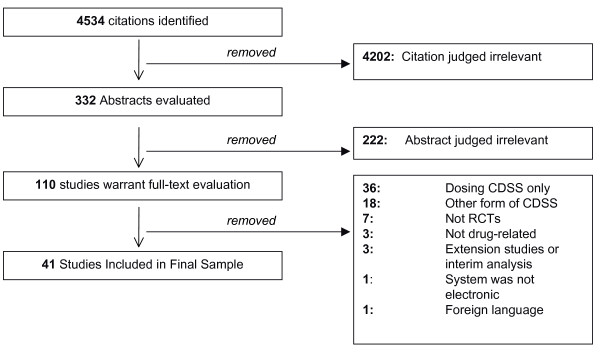
**Study flow diagram**. This diagram details the flow of citations through each stage of this systematic review.

Our search protocol returned 4534 unique citations (1179 from Medline, 1072 from EMBASE, 1053 from CINAHL, and 1204 from INSPEC plus an additional 26 from the reference lists). Of these, 332 abstracts were evaluated, and 110 were chosen for full text review (see study flow diagram in Figure 2). At this stage, 33 (30%) were removed for not meeting initial inclusion criteria (18 did not deal with prescribing, 7 were not randomized controlled trials, 3 were not drug-related, 3 were extension studies or interim analysis, 1 was a foreign language study, and 1 did not use a computer to offer the decision support). In addition, 36 (32.7%) were deemed to be a drug dosing CDSS and were excluded. The final review sample consisted of 41 studies (see Additional File 4) [9,21-60].

Key ratings, such as study quality and successful implementation showed good agreement, with kappa estimates of 0.62 (95% CI 0.50–0.73; weighted) and 0.77 (0.48 – 1.00) respectively. However, the features in the individual studies were more difficult to rate (see Additional File 5). Although the proportion of agreement for the presence of a feature was substantial, varying from 58.1% to 93%, the nature of the kappa statistic resulted in scores such as for 'CDSS supports the user's task at hand' at 0.14 (95% CI -0.18 to 0.46). Ultimately, the consensus rating for each variable was used for final data analysis.

### Description of Studies

The 41 RCTs involved a total of 612,556 patients (range 169–407,460 per study) and 2963 providers (range 17–334 per study). The mean methodological score was 5.9 with a range of 2–8, indicating generally good quality studies. Twenty-three studies (56.1%) used a RxCDSS in an outpatient general practice or internal medicine setting, 10 (24.4%) in inpatient hospital wards or emergency rooms, 5 (12.2%) in pharmacies and 3 (7.3%) in specialty clinics (2 for paediatrics and 1 for diabetes). The systems addressed a variety of problems – cardiovascular care (36.6%), general/internal medicine (29.3%), diabetes (9.8%), respiratory disease (9.8%), otitis media (7.3%), depression, osteoporosis and infectious disease (2.4% each). Nineteen (46.3%) of the RxCDSS were integrated with drug order entry, 16 (39.0%) with management/electronic health record (EHR) software and 9 (22.0%) also printed the suggestions. While 20 (48.8%) of these systems appeared to be developed by independent vendors, 21 (51.2%) were developed within an (experienced) home EHR environment such as the Regenstreif Medical System [61] and the Veterans Health Information System [62].

The studies most often employed a traditional 2-arm parallel design (31, or 75.6%), although 6 studies (14.6%) used a 3-arm design and 4 (9.8%) used a 2 × 2 factorial design. The nature of the control arm of studies was also mixed but most often was labelled as usual care (35 or 85.4%). The remaining 6 studies employed a control group intervention that was felt to be much less effective such as distribution of general treatment guidelines or education, reminders regarding an unrelated condition or a RxCDSS that operated with reduced functionality.

Systems were successfully implemented in 37 trials (90.2%) [21-23,25-30,32,33,35-60], provider behaviour changed in 25 (61%) [21-23,25,26,28,29,32,33,35,37,41,43-45,47,48,50,51,53,55-57,59,60], and patient outcomes improved in 5 (12.2%) [28,44-46,59]. Only 23 RCTs reported on this important outcome and all of the studies reporting success with a patient outcome were published after 2005. Of those studies reporting improvements in patient outcomes, Kucher *et al*. [44] noted a computer program encouraging prophylaxis against deep-vein thrombosis and pulmonary embolism reduced the risk of these two events by 41% at 90 days (P = 0.001). Javitt *et al*. [28] found a system that scanned administrative claims and clinical data to detect physician errors reduced hospital admissions by 19% in the intervention group relative to the control group (P < 0.001). Feldstein *et al*. [59] used patient-specific guideline reminders within the primary care physician's EHR to improve osteoporosis management for HMO patients who had suffered a previous fracture. Lester *et al*. [45] used e-mail reminders to physicians to encourage the increased use of lipid-lowering medication for patients with coronary artery disease. Roumie *et al*. [46] examined strategies to improve blood pressure control and found that patient education combined with computerized patient-specific alerts to providers was superior to provider education alone.

### Association Between CDSS Features and Outcomes

These associations are shown in detail in Additional File 1. Overall, studies mentioned an average of 17 features each (see Additional File 6). No trial concentrated on the features and their relationship with success or failure of the intervention nor did any trial systematically identify all of the features of their intervention. Only one study [21] isolated a specific RxCDSS feature through their randomization procedure. In this study, no benefit was found by adding bibliographic citations to electronic reminders. Another study [42] randomized two groups to guideline-based suggestions for treating congestive heart failure versus these suggestions plus others based on symptoms gleaned from the linked EHR, and found that the intervention group fared worse in terms of more hospitalizations. As this study did not have a control arm without a RxCDSS, it was not considered further in the analysis of features.

Because of the small number of studies, the lack of rigorous attention to features descriptions by the trials and, especially, the lack of diversity of outcomes across the 3 domains, we were unable to statistically evaluate whether there are features more associated with success than failure using logistic regression as originally planned. In general, the features most prevalent across the 40 remaining studies included: support of the user's task at hand (95%), provision of decision support at the time and place of decision-making (85%), provision of a recommendation rather than just an assessment (85%), automatic provision of decision support as part of clinician workflow (78%), integration with charting or order entry (75%), and convenient locations for the computers (68%). However, with few exceptions, the prevalence of these features was similar between successful and unsuccessful studies when examining implementation, provider behaviour and patient outcomes. For patient outcomes, there was reasonable separation for the prevalence of a few features. The features which all of the successful studies [28,44-46,59] shared and which most of the unsuccessful studies did not, included: provision of a recommendation rather than just an assessment, justification of decision support via provision of research evidence and the system uses data standards that support integration. However these results should be considered hypothesis-generating at best, given the low number of studies which successfully altered patient outcomes and the poor general reporting of features.

## Discussion

We have systematically reviewed the literature surrounding RxCDSS. The distribution of success in these 41 studies – the majority successfully implemented, more than half reporting changes in provider process but only five were able to successfully impact patient-related outcomes, appears to validate our hierarchal definition of success. The primary finding of the review is the continued poor reporting of and, by implication, the poor attention to system design and implementation features [12]. The lack of rigorous attention to and reporting of intervention features severely hampers progress in this field. All CDSS are by definition, complex interventions meaning mutifactorial, multidisciplinary and usually multi-staged [20,63].

If the ideal set of features was known, these could be highlighted and those more likely to be wasteful of time and resources could be dropped. For example, an activity with enormous cost in time and effort such as training and support of users, would rapidly change if high quality evidence suggested that only selected components and timing were the key to success.

The small number of trials and the lack of consistent reporting of features in the individual studies prevented statistical analysis of associations of features with outcomes. The descriptive examination of feature prevalence and their association with success versus failure returned no clear message.

The strengths of this review include a detailed search protocol tailored to four individual databases, the explicit use of a comprehensive features list, and a multi-level evaluation of system success. However, our study was limited, as mentioned, by the small sample size of included studies and the lack of systematic reporting of system features. Publication bias is always a possibility and is difficult to refute – in this case there may be an under-representation of negative studies. Many studies were excluded because the interventions dealt only with drug dosing suggestions. This group of studies should be systematically reviewed separately; it may well be that the simplicity of dosing decision support is an easier area to build success than complex processes of connecting diagnosis with therapy in light of contraindications, allergies and co-medications.

Despite the substantial interest and investment in developing electronic decision aids [64-68], our review supports the results of others who have noted a lack of demonstrated impact on clinically important patient outcomes [11-13]. Only 23 studies reported on patient outcomes; of these, 5 were successful. In the limited literature that evaluated such endpoints, patient outcomes were frequently secondary outcomes, with resultant lack of power to detect a difference between the intervention and control groups. This speaks to the lack of mature research programs in this field as well as the difficulties organizing and completing these difficult, complex intervention trials.

## Conclusion

This systematic review suggests that electronic prescribing decision support systems can be implemented and have the potential to change clinician behaviours, but there is no consistent translation into improved patient outcomes. We have demonstrated that trials do not adequately report and may not give sufficient attention to features of their system design and implementation. We believe that the lack of attention to evidence-based optimization of RxCDSS interventions continues to hamper the development and implantation of these essential systems.

## Competing interests

The authors declare that they have no competing interests.

## Authors' contributions

BM and JJRC were involved in all aspects of study design, literature search, data extraction, descriptive statistical analysis, and manuscript preparation. AMH was responsible for the study's conception and funding, participated in its design and execution, helped to draft the manuscript and revisions. MS was involved in review and renovation of the manuscript and led the latest update of results. LT was involved in the design of the statistical analysis and aided in the interpretation of the results. GF was responsible for conducting the statistical analysis and aided in the interpretation of the results.

## Pre-publication history

The pre-publication history for this paper can be accessed here:

http://www.biomedcentral.com/1472-6947/9/11/prepub

## Supplementary Material

Additional file 2 **Appendix A – Description of features thought to impact clinical decision support system success.** This table describes features which might impact success in clinical decision support systems, and provides references and examples for each. Click here for file

Additional file 3 **Appendix B – In-depth online search strategy.** This document provides a detailed protocol for the online search strategy utilized in this systematic review. Click here for file

Additional file 4 **Appendix C – Summary of studies included in the review.** This table summarizes the intention and outcomes of the randomized controlled trials included in this systematic review. Click here for file

Additional file 5 **Appendix D – Reliability scores when evaluating feature prevalence.** This table summarizes the intention and outcomes of the randomized controlled trials included in this systematic review. Click here for file

Additional file 1 **Table 1 – Prevalence of 28 features in 40 successful and unsuccessful randomized trials. **This table provides a detailed look at the prevalence of 28 RxCDSS features in 40 randomized controlled trials Click here for file

Additional file 6 **Appendix E – Detailed assessment of features from studies included in the review.** This table summarizes the reviewers' assessment of the prevalence or absence of features from studies included in this review. Click here for file
