# Supplementary material for: Features predicting the success of computerized decision support for prescribing: a systematic review of randomized controlled trials
Source: BMC Med Inform Decis Mak. 2009 Feb 11;9:11. doi: 10.1186/1472-6947-9-11 (PMC2667396; doi:10.1186/1472-6947-9-11)
Supplement: Additional file 3 — Appendix B – In-depth online search strategy. This document provides a detailed protocol for the online search strategy utilized in this systematic review. [file 1472-6947-9-11-S3.doc]

### Appendix B: In-depth Online Search Strategy

### Medline Search Protocol

Database: Ovid MEDLINE(R) <1966 to June Week 3 2008>

Search Strategy:

-----------------------------------------------------------------------

1 exp Artificial Intelligence/

2 exp Therapy, Computer-Assisted/

3 exp Decision Support Systems, Clinical/

4 exp Therapy, Computer-Assisted/

5 exp Medical Records Systems, Computerized/

6 exp Reminder Systems/

7 exp Hospital Information Systems/

8 exp Computer Systems/

9 exp Decision Support Techniques/

10 exp Ambulatory Care Information Systems/

11 exp Information Systems/

12 exp Decision Making, Computer-Assisted/

13 Software/

14 Computers/

15 1 or 2 or 3 or 4 or 5 or 6 or 7 or 8 or 9 or 10 or 11 or 12 or 13 or 14

16 exp Medical Errors/

17 exp therapeutic uses/

18 exp Drug Therapy/

19 exp Drug Information Services/

20 exp Drug Interactions/

21 exp Drug Monitoring/

22 exp Guideline Adherence/

23 exp Medication Systems/

24 exp Drug Administration Schedule/

25 exp drug costs/ or exp economics, pharmaceutical/

26 exp Dose-Response Relationship, Drug/

27 16 or 17 or 18 or 19 or 20 or 21 or 22 or 23 or 24 or 25 or 26

28 15 and 27

29 Drug Therapy, Computer-Assisted/

30 28 or 29

31 limit 30 to (humans and randomized controlled trial)

### EMBASE Search Protocol

Database: EMBASE <1980 to 2008 Week 25>

Search Strategy:

-----------------------------------------------------------------------

1 exp Artificial Intelligence/

2 exp Computer Assisted Therapy/

3 exp Decision Support System/

4 exp Reminder System/

5 exp Hospital Information System/

6 exp COMPUTER/

7 exp Computer System/

8 exp Computer Program/

9 exp information system/ or exp decision support system/

10 exp Medical Record/

11 1 or 2 or 3 or 4 or 5 or 6 or 7 or 8 or 9 or 10

12 exp Drug Therapy/

13 exp Drug Information/

14 exp Drug Interaction/

15 exp Drug Monitoring/

16 exp Practice Guideline/

17 exp Drug Administration/

18 exp "Drug Cost"/

19 exp PHARMACOECONOMICS/

20 exp dose response/ or exp drug dose/

21 exp Medical Error/

22 exp therapy/

23 12 or 13 or 14 or 15 or 16 or 17 or 18 or 19 or 20 or 21 or 22

24 11 and 23

25 exp Computer Assisted Drug Therapy/

26 24 or 25

27 limit 26 to (human and article)

28 Randomized Controlled Trial/

29 27 and 28

### CINAHL Search Protocol

Database: CINAHL - Cumulative Index to Nursing & Allied Health Literature <1982 to June Week 3 2008>

Search Strategy:

-----------------------------------------------------------------------

1 exp Artificial Intelligence/

2 exp Therapy, Computer Assisted/

3 exp Decision Support Systems, Clinical/

4 exp Health Information Systems/

5 exp Computer Systems/

6 exp Decision Making/

7 exp "Computers and Computerization"/

8 exp SOFTWARE/

9 1 or 2 or 3 or 4 or 5 or 6 or 7 or 8

10 exp Drug Therapy/

11 exp Drug Information Services/

12 exp Drug Interactions/

13 exp Drug Monitoring/

14 exp Practice Guidelines/

15 exp Medication Systems/

16 exp Drug Administration Schedule/

17 exp Economics, Pharmaceutical/

18 exp Dose-Response Relationship, Drug/

19 exp Treatment Errors/ or exp Medication Errors/

20 10 or 11 or 12 or 13 or 14 or 15 or 16 or 17 or 18 or 19

21 9 and 20

22 exp Clinical Trials/

23 21 and 22

### INSPEC Search Protocol

1. artificial intelligence OR computer aided analysis OR computer application OR computer software OR computerized monitoring OR decision support systems OR medical control systems OR medical expert systems OR medical information systems OR medical computing OR information systems
2. drugs OR drug delivery system OR pharmaceuticals OR pharmaceutical technology
3. 1 AND 2
4. Limit 3 to Journal Articles
